# Surgery in Munich; Prof. Nussbaum; Extirpation of Thyroid Gland

**Published:** 1878-09

**Authors:** 


[*From our Foreign Correspondent.*]

No. I.

### SURGERY IN MUNICH; PROF. NUSSBAUM; EXTIRPATION OF THYROID GLAND.

Muenchen, July 8, 1878.

*Editors Chicago Medical Journal and Examiner:*

Soon after my arrival in the city I called upon Prof. D. von Nussbaum to whom I had a letter of introduction. He received me with the greatest courtesy and has improved every opportunity to make my visit a profitable one. He is now the most prominent and most influential member of the medical faculty. He is about 50 years of age, medium sized and has fine features, with an expression of superior intelligence. He is not what might be called an orator, but a good lecturer, using plain language without the many technical expressions so common among German teachers. He gives a surgical clinic every day from 8 to 9 a. m. in the general hospital. He is an enthusiastic admirer of antiseptic surgery. With the exception of Lister, antiseptic surgery owes him the greatest debt of gratitude for its general introduction all through the German empire. His operations, with their results border almost upon the miraculous. Last week I witnessed an operation for the extirpation of a large sarcomatous tumor, situated in the dorsal region between and about the centre of the scapulae. The patient was a man about 50 years of age. He had been operated upon three times before; for the first time about 15 years ago, and the last time five years ago, the tumor recurring at variable times in the cicatrix. The patient is anæmic and somewhat emaciated, but no glandular infection can be ascertained. The tumor is somewhat elongated from above downwards, with several knotty red elevations near its superior border. It is apparently intimately connected with the deep fascia but not with the bones. Chloroform was administered. The whole operation was performed under a large spray from two spray producers. The skin over the tumor was included by two elliptical incisions. As the tumor was found to be very vascular and numerous large vessels passing into its interior, the base was divided into at least 25 pedicles with the ordinary dressing forceps, and each successively ligated with catgut and cut with the scissors. Very little blood was lost during the whole operation. The operator placed great stress on the importance of preventing great loss of blood in the aged, as it was very liable to be followed by serious if not fatal prostration. In closing the wound it was found that the skin had retracted to such an extent that proceeding in the ordinary manner the tension would be too great and would prevent primary union, a result so much to be desired in this case. To relieve the tension of the wound he applied what he terms the "Entspannungs-Naht." A large silver wire carrying needle is introduced about two inches from the margin through the whole thickness, and is brought out at about the same distance on the opposite side, carrying a stout silver wire; to the ends of the silver wire a disk of lead the size of a quarter is fastened. Three such sutures relieved the tension and rendered the accurate closure of the wound with catgut sutures possible.

A drainage tube was placed in the bottom of the wound and cut at both ends on a level with the surface. The wound was dressed according to Lister's directions, being covered by thin antiseptic silk, eight layers of moist antiseptic gauze, eight layers of dry gauze, with a layer of Mackintosh cloth between the last two layers; around the neck and lower part of chest the irregular spaces were filled with salicylic cotton, and the whole retained by a gauze roller. During his remarks, after the operation, the professor stated that he had repeatedly employed as many ligatures as in this case, and had never found them to cause suppuration or interfere with primary union. They do not act as foreign bodies, but are absorbed.

In speaking of the nature of the tumor he said he belonged to that class of pathologists who believe that benign tumors may after a great many years, assume a malignant type. To-day I was present at his eleventh operation for knock-knee, which consisted in sawing off the internal condyle of the femur subcutaneously, but as I wish not to weary your readers, I will reserve the report of this case for my next communication.

Very respectfully,

N. Senn.

---

### No. II.

Munich, July 15, 1878.

*Editors Medical Examiner and Journal:*

In my last letter I promised to describe an operation for knock-knee performed by Prof. von Nussbaum July 7. The deformity depends essentially upon an excessive development of the internal condyle of the femur. The inner portion of the articular surface of the femur being lower than when the relations of the condyles are normal, the leg is turned outwards proportionate to the extent of abnormal projection of the internal condyle. This fact led Oxdorn to separate the internal condyle of the femur subcutaneously, sliding it upwards ; in this manner correcting the deformity. The patient was a young man about 30 years of age. The deformity had made its appearance during his early youth. Only one side was affected. The abduction of the leg was so marked as to interfere seriously in locomotion. A small incision was made about eight centimeters above the joint, and over the inner margin of the rectus femoris muscle. A large tenotomy knife was now introduced beneath the skin along the inter-condyloid fossa to the inner margin of the tendon of the patella, and by dividing the soft parts down to the bone a tunnel was made for the saw. The saw used was Adam's, with a cutting edge two inches in length, blunt at the end, and a smooth shaft connecting it with a large firm handle. It was introduced along the tunnel in the same direction, and the bone divided from before backwards sufficiently until the bridge left could be fractured by forcible adduction of the leg ; this took place with an audible snap, when the leg could be easily placed in its natural axis. It is important not to complete the section with the saw, as the artery beneath might be injured, but to complete the operation by forcible manipulation. The opening in the skin was accurately closed with catgut sutures. Very little hæmorrhage took place. A well-padded splint was applied along the outer side of the leg and thigh, retained by a roller. The whole operation was performed under the antiseptic spray, and the wound covered by an antiseptic dressing. Directions were given that passive motion should be made in a few days. Prof. von Nussbaum has performed this operation eleven times ; in all the result was perfect, straight limb without anchylosis ; in none did suppuration take place. When we consider the utter inefficiency of all methods of treatment previously recommended for the correction of this deformity in advanced cases, and at the same time may be confident of the harmlessness of the procedure, we gladly welcome this operation as one of the greatest triumphs of orthopedic surgery.

### Extirpation of Thyroid Gland.

June 10, I received a private invitation to witness an operation for extirpation of a large goitre at his private hospital, located Nymphenburger strasse 28. The patient was Baroness X., about 50 years of age. She had been suffering from enlargement of the thyroid gland for many years. The suffering from the pressure of the tumor has gradually increased, until at present she is subject to frequent attacks of asphyxia. She is anxious to have the operation performed, even if attended with immediate risk to life, as it is the only means to afford possible relief. The tumor is quite firm, the size of a large fist, and located over the center of the trachea. Chloroform was administered by staff surgeon Bratsch. The head was thrown backwards by placing a pillow under the neck. An incision was made in the median line from the upper margin of the tumor to near the sternum ; on the right side another incision was connected with this, and carried along the upper margin of the clavicle to the external jugular vein. The skin was separated from the tumor, and a number of small veins ligated with catgut. On the surface of the tumor large veins were seen, some the size of the little finger, running in all directions.

The tumor was separated from the structures around and beneath it by making small pedicles with the dressing forceps, drawing through the openings made with the forceps, catgut ligatures, and after tying them, cutting the intervening portion with the scissors. Notwithstanding the great care exercised to prevent hæmorrhage, it at times became alarming, from slipping of the ligatures on the surface of the tumor, and from division of vessels not included in the ligature. Several times the pulse became imperceptible, and respiration almost suspended, but the greatest difficulty met with was at the base of the tumor, where it was intimately connected with the larynx and trachea ; the walls of the larynx and trachea had become extremely thin from the long continued pressure of the tumor, and in attempting to separate it the trachea was opened, blood immediately entered, and respiration ceased. An elastic catheter was introduced into the trachea, and the blood withdrawn by the mouth of the operator ; artificial respiration made by compressing the thorax.

As soon as respiration became re-established, a double tracheotomy tube was introduced, and the operation completed. The operation lasted over an hour, and but for the presence of mind of the operator, and his great courage, as well as the skillful assistance rendered by such men as Bratsch, Lindpainter and Messer, the patient would certainly have died on the table. Forty-six catgut ligatures were used. After all hæmorrhage had ceased, the wounds were closed, and the trachea tube secured with a tape tied around the neck. As a dressing, borax lint was used. The patient rallied after application of a strong current of electricity ; respiration became regular. She was placed in bed, and external heat applied. Nourishment was introduced by means of a stomach tube.

The patient died 24 hours after operation.

Yours truly,

N. SENN.

